# A comparative, epidemiological study of acute renal colic presentations to emergency departments in Doha, Qatar, and Melbourne, Australia

**DOI:** 10.1186/s12245-017-0160-9

**Published:** 2018-01-03

**Authors:** Sameer A. Pathan, Biswadev Mitra, Zain A. Bhutta, Isma Qureshi, Elle Spencer, Asmaa A. Hameed, Sana Nadeem, Ramsha Tahir, Shahzad Anjum, Peter A. Cameron

**Affiliations:** 1 Emergency Department, Hamad General Hospital, Hamad Medical Corporation, P.O.BOX 3050, Doha, Qatar; 2 School of Public Health and Preventive Medicine, Monash University, Melbourne, Australia; 3 National Trauma Research Institute, The Alfred Hospital, Melbourne, Australia; 4 Emergency & Trauma Centre, The Alfred Hospital, Melbourne, Australia

**Keywords:** Kidney stones, Urolithiasis, Nephrolithiasis, Renal colic, Epidemiology

## Abstract

**Background:**

This study aimed to compare the epidemiology, clinical presentations, management, and outcomes of renal colic presentations in two major academic centers from geographically diverse populations: Qatar (a country in the Afro-Asian stone belt) and South-Eastern Australia (not within a stone belt).

**Methods:**

We undertook a retrospective cohort study of patients with renal colic who presented to the Hamad General Hospital Emergency Department (HGH-ED), Qatar, and The Alfred ED, Melbourne, Australia, during a period of 1 year from August 1, 2012, to July 31, 2013. Cases were identified using ICD-9-CM codes, and an electronic template was used to record the data on predefined clinical variables.

**Results:**

A total of 12,223 from the HGH-ED and 384 from The Alfred ED were identified as renal colic presentations during the study period. The rate of renal colic presentations at the HGH-ED was 27.9 per 1000 ED visits compared to 6.7 per 1000 ED visits at The Alfred ED. Patients presenting to the HGH-ED were significantly younger [34.9 years (29.0–43.4) than The Alfred ED [48 years (37–60); *P* < 0.001].

The median stone size was larger in the HGH-ED group [6 (4–8) mm] versus The Alfred ED group [4 (3–6) mm, *P <* 0.001]. The intervention rate in the stone-positive population was significantly higher in the HGH-ED group as opposed to The Alfred ED group (38.7 versus 11.9%, *P* < 0.001). At the time of discharge, The Alfred ED group received fewer analgesic prescriptions (55.8 versus 83.5%, *P <* 0.001) and more tamsulosin prescriptions (25.3 versus 11.7%, *P <* 0.001).

**Conclusions:**

Renal colic presentations to the HGH-ED, Qatar, were younger, with larger stone size mostly located in the lower ureter, compared to The Alfred ED, Melbourne, Australia. The findings suggest that the benefits of treatment including medical expulsion therapy will vary between the two populations. Differences in epidemiology and patient mix should be considered while tailoring strategies for effective management of patients with renal colic in a given setting.

## Background

Urolithiasis usually presents as renal colic to the emergency department (ED) with agonizing pain. Renal colic is a clinical presentation of acute onset of flank pain, mostly in young adults, often with radiation to the groin, as well as hematuria and dysuria. The reported prevalence of renal colic varies significantly from 5 to 15% based on the geographical distribution of the disease [1]. Recurrence is common being up to 50%, and the incidence of renal colic is higher in the male population. The cost associated with urolithiasis is significant, reaching up to $6 billion in the USA, where it results in more than a million visits to EDs annually [1].

Stone belts, at a regional and global level, have been described, based on a high prevalence of urolithiasis in a particular population and geographical region. Populations in the western hemisphere (5–9% in Europe, 12% in Canada, 13–15% in the USA, mostly southeastern region) have been reported to have a higher risk of developing urolithiasis than the eastern hemisphere (1–5%) [2, 3]. The Afro-Asian stone belt [4], described as extending from Egypt, Sudan, Kingdom of Saudi Arabia, United Arab Emirates, Kuwait, Iran, Thailand, India, Pakistan, and the Philippines, has been reported to have a prevalence varying from 4 to 20% [5].

Although regions with a high frequency of presentation for renal colic represent ideal study sites for interventions, the differences in epidemiology when compared with low-frequency regions, may alter the generalizability of results. While meta-analyses of studies examining medical expulsion therapy (MET) appear to support alpha-blockers, a high-quality randomized controlled trial, enrolling patients from one geographic region, failed to validate the benefits of MET in that study population [6]. Evidence favoring intramuscular NSAIDs as being the more effective analgesic for renal colic has recently been published from a population in the Middle East, incorporating a significant proportion of young patients of Southeast Asian ethnic origin [7, 8]. Whether this can be generalized to different health systems with different ethnic populations is unclear. Further information about population and stone characteristics observed in the Afro-Asian stone belt will help to assess the applicability of evidence to and from this population.

This study aimed to describe the incidence, epidemiological features, clinical presentations, management, and outcomes of renal colic presentations to an ED in Qatar, a country in the Afro-Asian stone belt, and compare this to a population from Melbourne, Australia, not within a stone belt.

## Methods

### Study design and settings

This was a retrospective observational study of acute renal colic presentations to two major EDs. Cases were identified using ICD-9/10-CM codes for renal colic from ED attendance registries. The ED at Hamad General Hospital (HGH-ED) in Doha, Qatar, has an annual census of approximately 470,000 that serves as the only tertiary-care ED in a country with a population of 2,700,000. The HGH is the major academic center and teaching hospital in Doha Qatar, and the ED presentations reflect the population of Qatar. The Alfred ED is a major adult quaternary referral center and has an annual census of approximately 60,000 patients per year. Urology consultation and services were present at both centers at all hours. Computed tomography (CT) imaging was available at both the centers round the clock. The practice at The Alfred ED was similar to most developed countries and included a CT non-contrast study for most of the patients presenting with renal colic [9]. There were pre-defined protocols for the imaging in renal colic at the HGH-ED during the study period, dictating selective imaging among patients at a high risk of complications. A CT scan was requested in the ED only for higher risk patients such as those with a single kidney, renal transplant, known renal impairment, having multiple comorbid conditions, frequent visits to the ED for similar pain within the last 2 weeks, signs of renal injury or sepsis, or patients with persistent pain not responding to parenteral analgesia. For females in reproductive age group, ultrasound was the initial imaging test at the HGH-ED. The limited CT and ED resources, a high volume of ED renal colic presentations in an otherwise healthy young population, were the main contributors to selective imaging approach at the HGH-ED.

### Participants and data collection

The study period chosen was August 1, 2012, to July 31, 2013. A pre-piloted Microsoft office Access 2007 electronic version form was used to extract the data by explicit chart review. The patients included in the study were aged 18 years and above. Variables included were patient demographics, ED presentation and triage priority assigned, investigation results (urine microscopy, blood cell counts, serum chemistry, and the CT or USG imaging results), treatment at the time of discharge and disposition from ED. Stone characteristics such as size, location, and the presence or absence of hydronephrosis, hydroureter, or fat stranding were extracted from diagnostic imaging reports. The maximum dimension of stone reported was recorded in millimeters. Patient medical records and the urology procedure registers were checked for 30 days from the index visit to ED or from the date of stone detection on imaging.

### Statistical analysis

All data were initially collected through Microsoft Access 2007 (Microsoft Corporation, USA), then imported to Stata 12.1 (College Station, TX 77845, USA). The Shapiro-Wilk or the Shapiro-Francia tests was used to test for normality as appropriate. The Student’s *t* test or the Wilcoxon rank-sum test was used to analyze the statistical significance of differences, as appropriate. A two-sided *P* value of less than 0.05 was considered to indicate statistical significance. Continuous outcomes are reported as mean (SD) for normally distributed data, and median (IQR) is reported for non-parametric data. Categorical outcomes are presented as proportions with 95% confidence interval.

### Ethics approval

The Alfred Research and Ethics Committee and Monash University Human Research Ethics Committee proposal number CF15/4323 - 2015001863 and Medical Research Center, Hamad Medical Corporation, proposal number 13380/13 approved the study, granting a waiver of consent from patients to extract information from medical records.

## Results

### Patient characteristics

During the study period, from August 1, 2012, to July 31, 2013, total ED visits were 438,100 to the HGH-ED and 57,313 to The Alfred ED. Prevalence of renal colic among the ED attendance population was 27.9 per 1000 visits (95%CI, 27.4 to 28.4) in the HGH-ED and 6.7 per 1000 visits (95%CI, 6.0 to 7.4) in The Alfred ED. A total of 12,223 from the HGH-ED, and 384 from The Alfred ED, were identified as being due to renal colic. Most patients presenting to The Alfred ED group were from Oceania, whereas, those in the HGH-ED group were from South Asia (51.2%), West Asia (22.2%), and North Africa (19.9%). The median age of patients in the HGH-ED group was 34.9 years (29.0–43.4), significantly lower than those in The Alfred ED group at 48 years (37–60) (*P* < 0.001). In both centers, most patients were assigned priority three at triage (Table 1).

**Table 1:** Group characteristics

Variables	HGH-ED, Qatar	Alfred ED, Australia	*P* value
Total renal colic clinical presentations, *n* (%)	12,223 (97.0)	384 (3.0)	
Age (years)	34.9 (29.0–43.4)	48 (37–60)	*P <* 0.001
Male, *n* (%)	10,270 (84.0)	279 (74.4)	*P <* 0.001
Priority assigned at triage, *n* (%)			
1	11 (0.1)	1 (0.4)	
2	1384 (11.3)	52 (18.1	
3	10,819 (88.5)	173 (60.3)	
4	9 (0.1)	61 (21.3)	
Hematuria, *n* (%)			
Yes	446 (67.7)	180 (76.0)	*P =* 0.017
Total	659 (100)	237 (100)	
Serum creatinine (μmol/L)	87 (71–106)	89 (75–109)	*P =* 0.026
Blood urea nitrogen (mmol/L)	4.9 (3.9–6)	6 (4.7–7.4)	*P <* 0.001
Total white cell counts, (×1000/μL)	9.4 (7.4–11.8)	10.5 (8.3–13.2)	*P <* 0.001
USG examination, *n* (%)	779 (6.4)	33 (8.6)	*P =* 0.144
CT scan (kidney, ureter, and bladder)	2678 (21.9%)	268 (69.7%)	*P <* 0.001
Overall imaging in the ED, *n* (%)	3003 (24.6)	278 (72.4)	*P <* 0.001
Stone detected on imaging, *n* (%)			
Found	2692 (89.6)	250 (89.9)	*P =* 0.882
Total	3003 (100)	278 (100)	
Intervention, if stone present, *n* (%)			
Performed	829 (30.8)	33 (13.2)	*P <* 0.001
Total	2692 (100)	250 (100)	
Analgesia at discharge, *n* (%)			
Prescribed	8524 (83.5)	183 (55.8)	*P <* 0.001
Total	10,206 (100)	328 (100)	
Tamsulosin at discharge, *n* (%)			
Prescribed	1296 (11.7)	83 (25.3)	*P <* 0.001
Total	11,076 (100)	328 (100)	

### Investigations in the ED

Overall, hematuria on urine examination was absent in 270 (30.1%; 95% CI, 27.1–33.3) of 896 patients. A significantly higher proportion of patients in The Alfred ED group had blood examination performed in the ED (87.2%) compared to the HGH-ED group (28.7%; *P* < 0.001). Among patients who had blood examination in the ED, the proportion of patients with abnormal creatinine levels, defined as > 110 μmol/L in males and > 90 μmol/L in females, were not significantly different (22.9% in HGH-ED vs 25.7% in Alfred ED; *P* = 0.257). The incidence of leukocytosis, defined as white blood cell counts > 10 × 1000/μL, was significantly higher in The Alfred ED group (56.8%) compared to the HGH-ED group (42.7%; *P* < 0.001). The use of ultrasonographic (USG) examination was low at 6.5% (95%CI, 6.0–6.9). The rate of performing non-contrast CT scans in The Alfred ED group (72.4%) was significantly higher than that in the HGH-ED group (24.6%; *P* < 0.001). Stone positive rates, in those patients who had an imaging test performed in the ED, were similar in the HGH-ED group (89.6%) and The Alfred ED group (89.9%, *P* = 0.882) (Table 1).

### Radiological findings

The median stone size detected on CT examination was larger in the HGH-ED group (6 mm; IQR, 4–8 mm) compared to The Alfred ED group (4 mm; IQR, 3–6 mm; *P* < 0.001). The overall incidence of renal stones was 35.7% (95%CI, 33.9–37.4) and ureteral stone was 57.5% (95%CI, 55.7–59.3). In the HGH-ED group, the most common stone locations were the lower ureter (26.7%) followed by the vesico-ureteric junction (22.4%). Among the patients presenting to The Alfred ED, the most common locations were the vesico-ureteric junction (35.7%) followed by the lower ureter (18.9%). The incidence of hydronephrosis was higher is the HGH-ED group (75.5%) compared to The Alfred ED group (60.4%, *P* < 0.001). The HGH-ED population had a higher incidence of multiple stones (28.3%) compared to The Alfred ED group (15.6%, *P* < 0.001) as detected on the CT examination (Table 2).

**Table 2:** Findings on imaging

Variables	HGH-ED, Qatar	Alfred ED, Australia	*P* value
Stone size (largest dimension), mm	6 (4–8)	4 (3–6)	*P <* 0.001
Number of stones, *n* (%)			
1	1347(51.9)	152 (60.8)	
2	417 (16.1)	44 (17.6)	
3	95 (3.7)	15 (6.0)	
Multiple	735 (28.3)	39 (15.6)	
Total	2594 (100)	250 (100)	
Location of stone, *n* (%)			
Renal pelvis calyx	466 (17.7)	27 (11.1)	
Pelvi-ureteric junction	42 (1.6)	16 (6.6)	
Upper ureter	434 (16.5)	43 (17.6)	
Mid ureter	211 (8.0)	18 (7.4)	
Lower ureter	704 (26.7)	46 (18.9)	
Ureterovesical junction	592 (22.4)	87 (35.7)	
Bladder	103 (3.9)	7 (2.9)	
Urethra	11 (0.4)	0 (0)	
Recently passed	75 (2.8)	0 (0)	
Total	2638 (100)	244 (100)	
Hydronephrosis, *n* (%)			
Present	2268 (75.5)	168 (60.4)	*P <* 0.001
Total	3003 (100)	278 (100)	
Hydroureter, *n* (%)			
Present	1844 (61.4)	133 (47.8)	*P <* 0.001
Total	3003 (100)	278 (100)	
Fat stranding, *n* (%)			
Present	1048 (34.9)	135 (48.6)	*P <* 0.001
Total	3003 (100)	278 (100)	

### Treatment

The rate of urological interventions was significantly higher in the HGH-ED group (30.8%) compared to The Alfred ED group (13.2%, *P* < 0.001). Most patients in the HGH-ED group were prescribed analgesia at the time of discharge (83.5%), and a smaller proportion was also prescribed the oral alpha-blocker, tamsulosin (11.7%). In contrast, there were more tamsulosin prescriptions in The Alfred ED group (25.3%; *P* < 0.001). The admission rates, among renal colic presentations, were 7.8% (95%CI, 7.4–8.3) from the HGH-ED and 16.4% (95%CI, 12.8–20.5) from The Alfred ED (*P* < 0.001).

## Discussion

Renal colic presentations to the HGH-ED, Qatar, were more common than that to an Australian ED. Patients presenting with renal colic in Qatar were younger and had a higher frequency of multiple stones. Median stone size observed in the Qatar population was larger, and most stones were found in the lower ureter or at the ureterovesical junction (UVJ). The observed differences in the investigations and management practices were partially explained by the high patient load and availability of limited resources. The findings will be utilized to apply current evidence and tailoring future management strategies.

Possible risk factors for stone formation studied in the past are commonly observed in Qatar. Which includes *the diet*, rich in animal protein, high oxalate, low calcium, and *the dry*, *subtropical desert climate*, leading to dehydration and causing concentrated urine [10–12]. These unique features may explain the high prevalence of renal colic in Qatar population and other Middle Eastern countries [13]. The observed high incidence provides a practical advantage for conducting large urolithiasis clinical trials in Qatar. The feasibility of conducting such trials has been tested through previous work on renal colic analgesia [13], and the results of this study may further help to assess the applicability of previously generated evidence in a population similar to Qatar.

The two study centers observed few variations in the assessment of renal colic patients in ED. Although imaging rates were significantly different between the groups, the stone detection rates over imaging were comparable to each other. In the Australian cohort, the stone detection rate was higher than previously reported rates from Australia [9]. The findings also suggest the possibility of missed stones among patients from the Qatar cohort who did not undergo CT scan imaging and indicate the need for close follow-up in such settings to avoid complications. However, the place of imaging, in ED versus outpatients, for a young, male population is debatable, especially considering the disease incidence, availability of resources, and the impact of urgent imaging on acute management [14]. Further research is warranted to assess the practice of selective CT scan imaging and its impact on patient-centered outcomes.

The larger stone size and frequent incidence of obstruction most likely contributed to higher surgical intervention rates in the HGH-ED renal colic population. In contrast, the use of MET was more in the Australian group that had smaller stone size most commonly seen at the UVJ. Such differences may have marked implications for the applicability of available evidence. As shown in meta-analyses and systematic reviews, renal colic due to stone size 5–10 mm in the lower ureter may benefit more from tamsulosin use by facilitating stone passage than the stones located higher in the urinary tract or with size out of this range [15, 16]. Therefore, we recommend increased use of tamsulosin for patients in Qatar who have larger stone size 5–10 mm in the lower ureter to get maximum benefits of MET [7, 8, 17].

Our results were consistent with previous studies published from the same geographical region. [18] However, in comparison with the published data from the Western world, some differences were observed in stone size, location, and the management of renal colic visits to ED. In Qatar, we observed the lower incidence of renal stones and higher rates of ureteral stones with hydronephrosis than centers in the USA [9, 14, 19, 20]. We also observed higher ED incidence, admission rate, and urological intervention rates in the population admitted from ED in Qatar than similar cohorts in the UK [2, 21]. The higher incidence of renal colic visits to ED can be a challenging and limiting factor for conducting an imaging exam for every patient during the ED visit whereas having most stones found in lower ureter may suggest that MET, ureteroscopy, and preventive measures may be more cost-effective approaches than other surgical modalities, for the management of urolithiasis in settings like Qatar [22]. However, tailoring of economic decision-tree model, for a safe diagnosis with effective treatment, and prospective validation of such are needed in the future.

### Limitations

This study is limited in analyzing data from two single centers. We were unable to collect all clinically relevant information such as chronic illnesses, dietary habits, environmental factors, and follow-up. Our conclusion about stone size in the Qatar population may have some selection bias as the CT imaging at HGH-ED was mostly performed in a population at risk of complicated stone disease. Finally, the true prevalence of renal colic cannot be accurately estimated as the prevalence measured in this study was per ED visit to single centers in the two countries. However, presentations to HGH-ED, Qatar, account for the majority of ED presentations for renal colic in the country, and renal colic presentations to The Alfred ED are expected to be representative of those presenting to other tertiary referral centers in Australia.

## Conclusions

Renal colic presentations to HGH-ED, Qatar, were younger, with larger stone size obstruction, mostly in the lower ureter, whereas at The Alfred ED, Melbourne, patients had smaller stone size and a comparatively lower incidence of obstruction. These differences should be considered while tailoring management approaches for patients with renal colic in a given setting.

## Abbreviations

CT: computed tomography
ED: Emergency department
MET: Medical expulsion therapy
NSAIDs: Non-steroidal anti-inflammatory drugs
USG: Ultrasonography
UVJ: Ureterovesical junction
